# Mating behavior and preliminary evidence of putative odor-mediated interactions in *Raoiella indica* (Acari: Tenuipalpidae)

**DOI:** 10.1007/s10493-026-01111-4

**Published:** 2026-03-08

**Authors:** Maria Isabel de Oliveira Lopes Gomes, Rhaisa Chaves dos Santos, José Wagner da Silva Melo, Debora Barbosa de Lima

**Affiliations:** Department of Zoology, Centro de Biociências, Programa de Pós-graduação em Biologia Animal, Universidade Federal de Pernambuco, Av. Professor Moraes Rego, 1235 - Cidade Universitária, Recife, 50670-901 PE Brasil

**Keywords:** Mating, Behaviour, Mite, Copulation, Cues

## Abstract

Odour and contact chemical traces play central roles in the sexual communication of mites, mediating partner encounter and recognition of the female reproductive status. We investigated the mating behavior of *Raoiella indica* Hirst and the role of female-associated chemical cues in male attraction. Behavioral observations were conducted under controlled laboratory conditions, and three complementary bioassays were performed: (i) detailed recording of courtship and copulation sequences, (ii) two-choice tests to evaluate male preference for arenas associated with females at different reproductive stages, and (iii) assays testing male responses to substrate-borne traces left by females. Our objective was to describe the mating sequence of *R. indica* and assess how odor and contact traces contribute to male. Mating followed a stereotyped sequence composed of four stages. Males showed preference only for arenas associated with teleiochrysalis females, whereas no preference was observed for virgin or mated adult females. In contrast, males were attracted to traces left by virgin females but not to those left by recently mated females. These results indicate that male orientation in *R. indica* is mediated by stage-specific chemical cues and that contact traces provide reliable information about female reproductive status. Overall, our findings expand knowledge of chemical communication and mating strategies in *R. indica*, offering new insights into the behavioral ecology of tenuipalpid mites.

**Supplementary Information:**

The online version contains supplementary material available at 10.1007/s10493-026-01111-4.

## Introduction

Reproductive behavior is one of the main determinants of population dynamics, geographic distribution, and ecological interactions among species (Anholt et al. 2020; Xu et al. 2023). Among arthropods, especially in groups with limited visual and auditory communication, such as mites, specific mating strategies have evolved under strong sexual selection pressure. These strategies include efficient mate location, recognition of reproductive status, and behaviors such as pre-copulatory guarding (Cone et al. 1971a; Rasmy and Hussein 1994; Oku et al. 2005; Oku 2010). One of the most widely used mechanisms in this context is chemical communication, either through the release of sex pheromones or the deposition of chemical cues on the substrate (Rasmy and Hussein 1994; Oku et al. 2005; Oku et al. 2015), which not only facilitates encounters between sexes but also optimizes male reproductive investment by directing them preferentially toward virgin or receptive females.

Mites may also rely on substrate-borne chemical trails deposited by females, in addition to volatile pheromones. These contact cues can inform males about the presence and reproductive status of potential mates, allowing orientation even when females are not directly visible. Such trails operate at shorter distances and complement volatile signals, increasing mate-finding efficiency and reducing the costs associated with courting non-receptive females (Oku et al. 2005; Rodrigues et al. 2017). Therefore, considering both volatile and trail-mediated communication is essential to understand how mating strategies are shaped in mites.

In several mite species, immature females in the quiescent stage release volatile compounds that guide males in locating potential mates (Cone et al. 1971a; Oku et al. 2009). This process can trigger male–male competition for copulation, ultimately leading to the establishment of pre-copulatory guarding, a behavior in which the male remains beside or on the female until she reaches sexual maturity (Goshima et al. 1998; Oku et al. 2015; Oku 2016). Pre-copulatory guarding is considered an adaptive strategy to maximize paternity success, particularly in reproductive systems where females are receptive for only a short period or where the first male to mate gains an advantage in sperm competition (Ridley 1983; Yasui 1988). This type of behavior is well documented in species of the families Tetranychidae and Tenuipalpidae (Manglitz and Cory 1953; Haramoto 1966; Cone et al. 1971b; Penman and Cone 1972; Potter et al. 1976; Hoy et al. 2007).

The family Tenuipalpidae comprises phytophagous species of economic importance, whose reproduction occurs predominantly through arrhenotoky, a system in which virgin females produce only males, whereas mated females produce both sexes (Gerson 2008). Despite the agricultural relevance of several species within this group, the mating behavior of Tenuipalpidae remains poorly understood in the literature, which limits the comprehension of their reproductive strategies and the potential use of behavioral control tools.

*Raoiella indica* Hirst (Acari: Tenuipalpidae) is an example of a biological model within this context of scarce information on mating behavior in the family. This mite is an invasive species with an expanding distribution across the Americas, causing significant damage to palms, bananas, and heliconias, with considerable economic impacts (Melo et al. 2018; Barros et al. 2020). Previous studies suggest that *R. indica* males locate quiescent immature females and remain near them for up to two days, waiting for ecdysis to occur in order to mate (Hoy et al. 2007). However, to date, no detailed description exists of the reproductive behavior phases or of the role of chemical communication in this process. Therefore, the present study provides the first comprehensive description of the mating behavior of *R. indica*, characterizing the duration and sequence of its main phases, the stimuli involved in male attraction, and the behavioral patterns associated with the discrimination of female reproductive status.

## Materials and methods

The experiments were conducted at the Acarology Laboratory of the Federal University of Pernambuco (UFPE), under controlled conditions of 27 ± 1 °C, 75 ± 10% relative humidity, and a 12-hour photophase in a rearing room.

### Obtaining R. indica

Individuals of *R. indica* were obtained from naturally infested coconut palm (*Cocos nucifera* L.) leaflets collected on the UFPE campus. To obtain virgin males and females, individuals were collected at the protonymph stage, when sexual dimorphism can already be distinguished, allowing for sex separation prior to maturation. After collection, each individual was maintained in isolation until reaching the desired experimental stage.

**Fig. 1:**
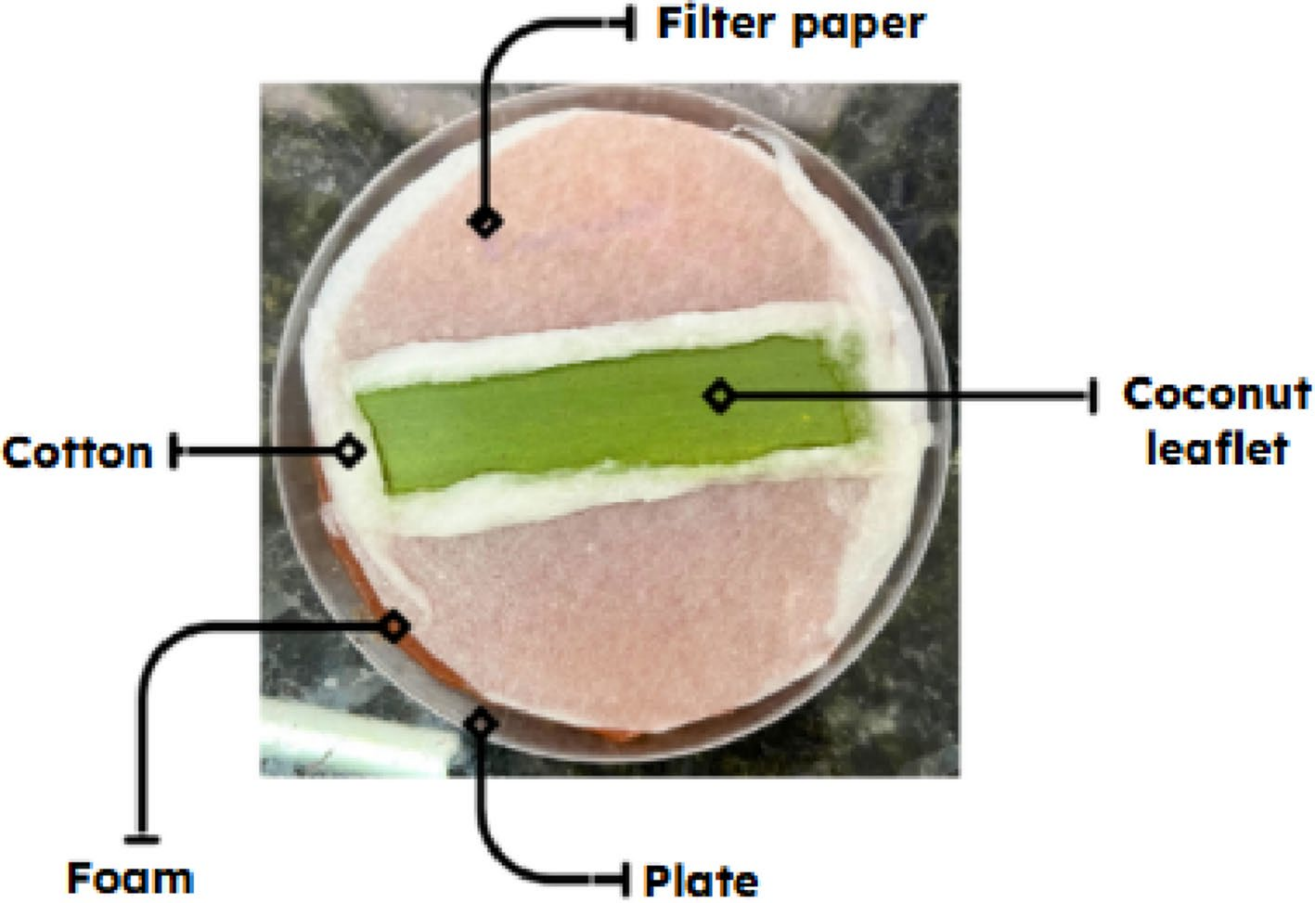
Confinement arena composed of a leaflet fragment of *C. nucifera* used to maintain individuals of *R. indica*

The mites were maintained in confinement arenas consisting of Petri dishes (9 cm in diameter) containing a polyethylene foam disc (8 cm in diameter × 1 cm thick) moistened with distilled water. A filter paper disc (7 cm in diameter) was placed on top of the foam, followed by a fragment of coconut leaflet (approximately 5 × 2 cm) The edges of the leaflet were covered with moistened absorbent cotton to prevent the escape of individuals (Fig. 1). All arenas were kept in a rearing room. *Raoiella indica* shows a clear preference for the abaxial (lower) surface of coconut leaflets. Therefore, observations and behavioral tests were standardized on this surface.

Three female conditions were used in the treatments: teleiochrysalis (the final developmental stage prior to adulthood), virgin adults, and mated adults. Virgin females were obtained by isolating teleiochrysalids until emergence, ensuring the absence of copulation. Mated females were obtained by pairing teleiochrysalids with virgin males, which were kept together until copulation occurred; females were considered mated 48 h after the separation of the mite pairs. In all treatments, virgin males approximately one day old were used.

### Mating behavior

A virgin female, one day after ecdysis, was transferred to the experimental unit using a fine brush. Subsequently, a virgin adult male, also one day old, was introduced. The mite pairs were observed for up to four hours under a binocular stereoscopic microscope, and the mating behavior was described in four distinct stages: exploratory contact, pre-copulatory position, genital position, and copulation.

For the analysis of mating behavior, 38 trials were conducted, of which 23 were considered valid. The discarded trials corresponded to cases in which no interaction occurred between male and female within the observation period, in which case both the experimental unit and the pair were eliminated. Each experimental unit corresponded to one trial and consisted of an arena similar to that described in the section “Obtaining *R. indica*”, but with the leaflet cut into a square shape measuring 2 × 2 cm. In the 23 valid trials, the latency to the onset of mating (time between the introduction of the male and the beginning of the behavior), the duration of each stage of the behavioral sequence, and the number of males that progressed through each phase of mating were recorded.

### Attraction of males to odours released by females

**Fig. 2:**
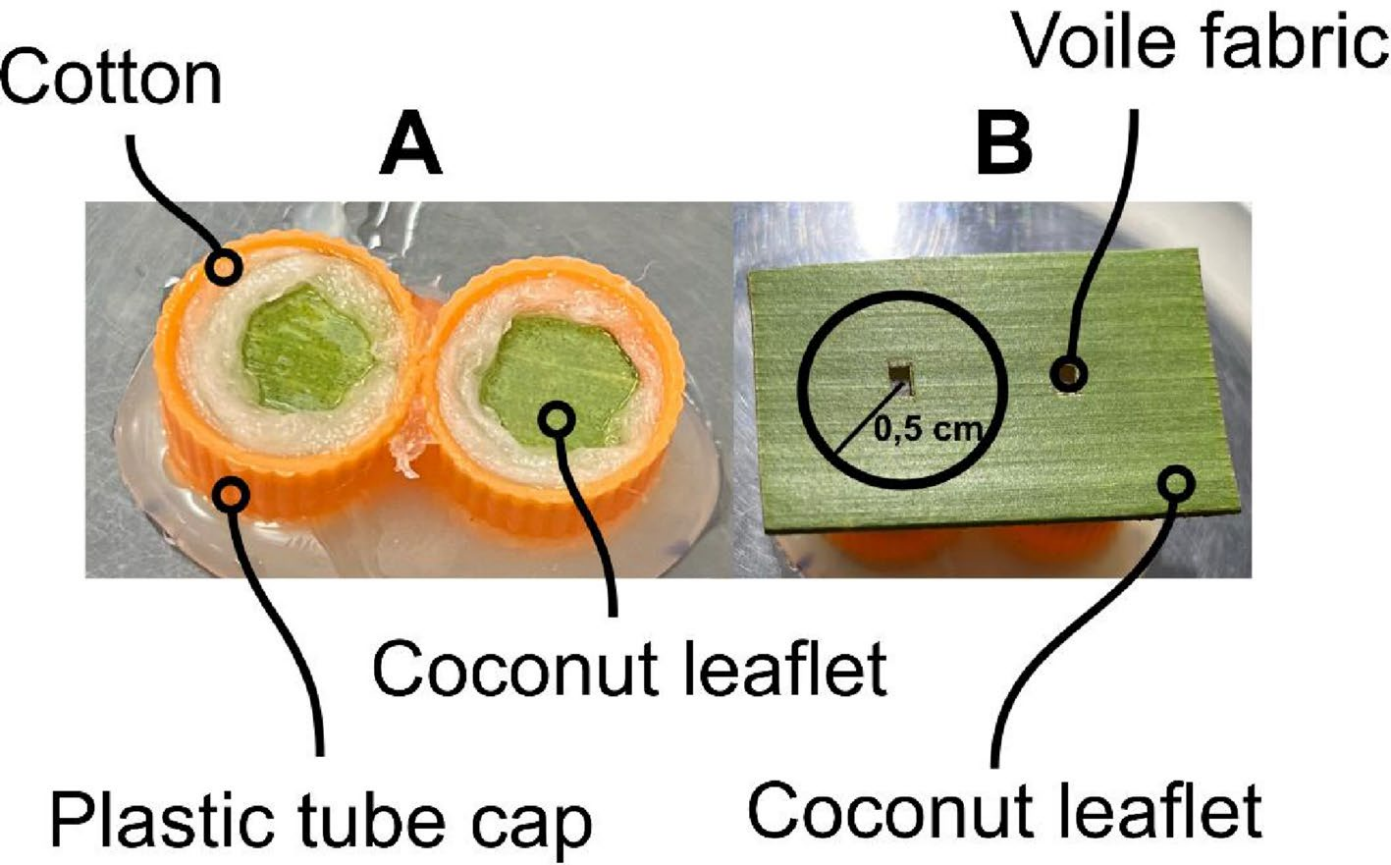
Experimental unit consisting of cages (**A**) and a leaflet fragment with openings covered with voile fabric (**B**). A male was considered to have made a choice when positioned within 0.5 cm of the voile-covered opening, indicated by the circle (**B**)

The experimental unit consisted of two plastic test tube caps (1 cm in diameter × 1 cm in height), each containing a circular leaflet fragment of coconut palm (*C. nucifera*) measuring 0.7 cm in diameter. The fragments were placed at the center of the caps, surrounded by hydrophilic cotton moistened with distilled water to prevent mite escape. On top of these caps, a rectangular leaflet fragment (2.5 cm × 1.5 cm) with two openings covered with voile fabric was placed, forming a cage-like structure (Fig. 2A, B).

In the experimental treatments, twenty females at different reproductive stages (teleiochrysalids, virgin adults, or mated adults) were introduced into one of the cages, while the other was left empty. Virgin males were introduced individually, one at a time, and each male represented an independent replicate. Each male was released at the center of the rectangular leaflet fragment and observed for 10 min. A male was considered to have made a choice when it approached within 0.5 cm of a cage opening. The time spent in this area was also recorded.

Twenty replicates were conducted for each treatment, with each male considered an independent replicate. At the end of each replicate, the leaflet fragment containing the voile-covered openings was replaced with a new one. To verify the absence of directionality in the experimental setup, a blank test was performed in which odours from 20 *R. indica* teleiochrysalis females were offered on both sides of the arena. In this test, ten trials were conducted.

### Male attraction to traces left by females

**Fig. 3:**
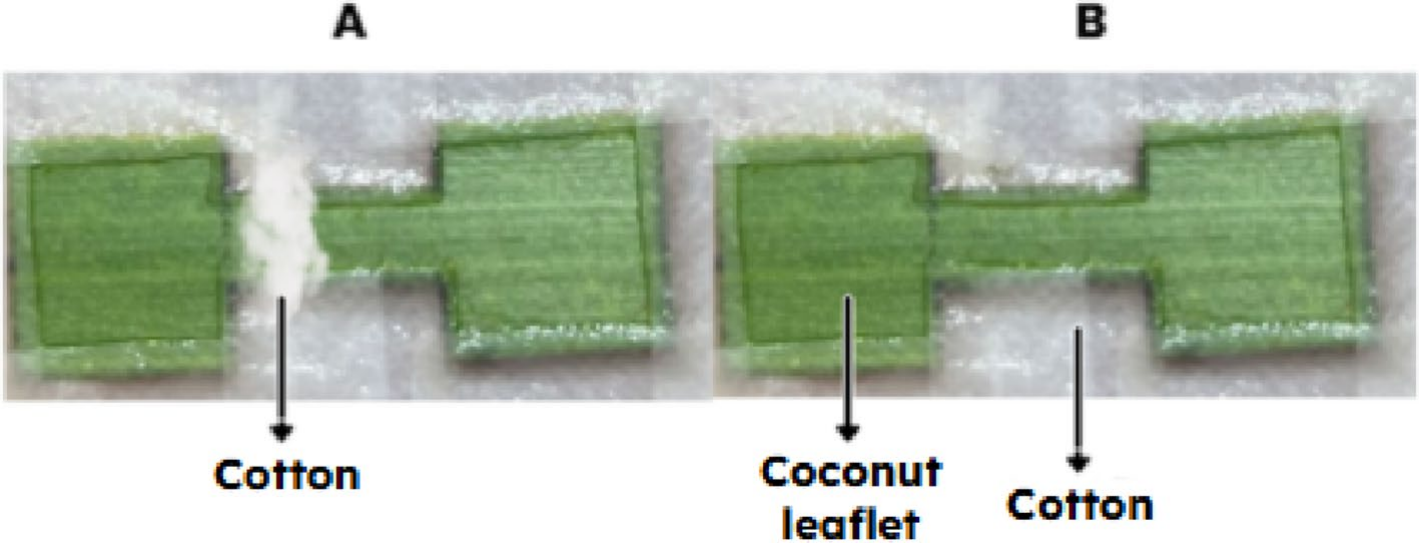
Dumbbell-shaped experimental unit: (**A**) before the start of the replicates, with cotton covering the central walkway; (**B**) during the replicates, after removal of the cotton from the central walkway

The experimental unit was similar to the confinement arena with leaflet described in the section “Obtaining *R. indica*.” However, the leaflet was cut into a dumbbell shape, with two square ends (1.5 cm × 1.5 cm) connected by a central walkway 1 cm long (Fig. 3A, B). The edges of the experimental unit were covered with hydrophilic cotton moistened with distilled water to prevent mite escape. To isolate the side containing female cues from the cue-free side, the central walkway was also covered with moistened hydrophilic cotton (Fig. 3a).

Chemical cues were generated by placing 20 virgin or mated adult females at one end of the arena for 24 h, allowing them to deposit possible secretions, excretions, and/or odors compounds. After this period, the females were removed from the experimental units using a fine brush, and the cotton covering the walkway was also removed. Subsequently, twenty previously isolated virgin males of *R. indica* were released at the center of the experimental unit. Male choice and the time spent at each end of the arena were recorded by direct observation for up to four hours. The time spent in the central walkway was not recorded and was not included in the analyses; only male behavior in the two terminal areas (with traces vs. without traces) was evaluated. At the end of each trial, both the leaflet fragment containing female cues and the male (each male representing one independent replicate) were replaced. To evaluate the non-directionality of the experimental arrangement, a blank test was performed in which traces left by 20 virgin adult females over 24 h were present on both sides. Ten replicates were conducted for this test. In this study, the term “contact chemical traces” is used to describe substrate-borne odor cues associated with females, which may derive from exuviae, feces or plant odors produced during feeding and tissue injury. The chemical identity of these cues remains unknown.

### Possibility of copulation between males and recently mated females

In a separate experiment, we evaluated whether males attempted to mate with females that had already copulated. The experimental unit was identical to that described in the “Mating behavior” section. A mated female (approximately three days after emergence), previously isolated from the confinement arena, was transferred to the unit using a fine brush. Subsequently, a virgin male, about one day old, was introduced from the confinement arena. Each pair was monitored under a binocular stereomicroscope for up to 24 h (represented the maximum evaluation period used to determine whether copulation occurred). Copulation was recorded when the male completed the final step of the mating sequence previously described in first experiment (see in “Mating behavior”). A total of 20 replicates were performed, with each experimental unit considered as one replicate.

### Statistical analysis

Statistical analyses of male choice for *R. indica* females and for the side of the coconut leaflet containing either a cage or chemical cues were performed using chi-square tests. Residence time data were analyzed using Student’s *t*-tests in SAS (SAS Institute 2008). Prior to analysis, homogeneity of variances was evaluated, and all tests indicated non-significant differences between variances (*p* > 0.05). Because the assumption of equal variances was satisfied, no data transformation was required and the pooled-variance *t*-test was used.

## Results

### Mating behavior

**Fig. 4:**
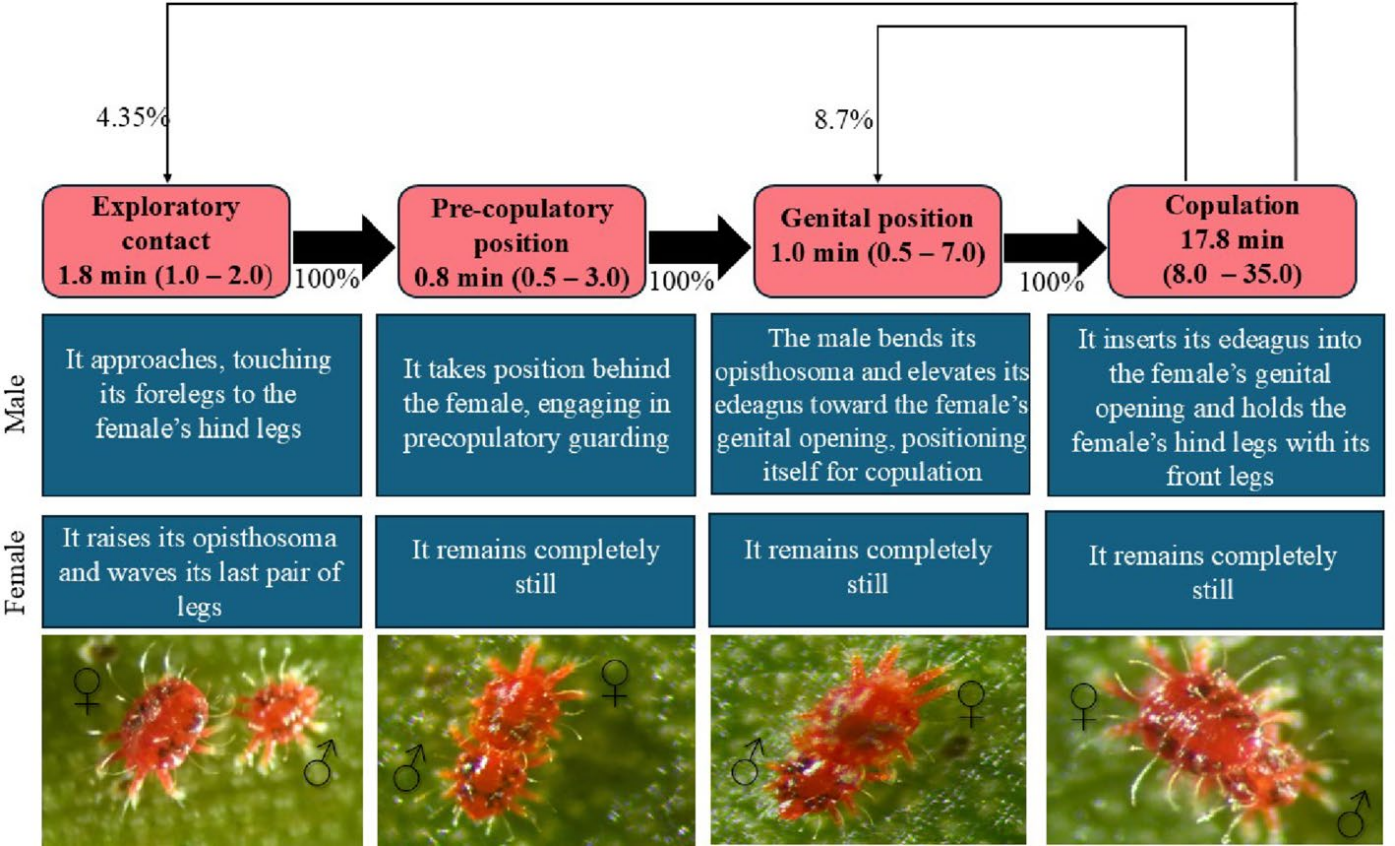
Ethogram of the mating behavior of *R. indica*, showing the different behavioral phases observed along with their respective frequencies of occurrence (%) and mean durations (min)

The mating behavior of *R. indica* was divided into four distinct stages. The mean latency for males to initiate the first contact with a female was 37.5 ± 7.09 min (*n* = 23). In the first stage, termed exploratory contact, males approached females by touching their anterior legs to the posterior legs of the females. Simultaneously, females raised the opisthosoma and moved their posterior legs. This stage lasted an average of 1.8 ± 0.08 min. The second stage, the pre-copulatory position, occurred when males positioned themselves behind the female, assuming a guarding posture while the female remained immobile. This stage lasted on average 0.8 ± 0.13 min (48 s). The third stage, the genital position, was characterized by the male bending the opisthosoma and raising the aedeagus toward the female genital opening, while the female remained immobile. This stage lasted an average of 1.0 ± 0.27 min. Finally, in the fourth stage, the copulation, males inserted the aedeagus into the female genital opening while simultaneously touching the posterior legs of the female with their anterior legs. Throughout this process, the female remained immobile. This stage lasted on average 17.8 ± 1.02 min, resulting in a total mean duration of 21.4 ± 1.45 min for the entire mating sequence, from exploratory contact to the end of copulation (Fig. 4). All observed pairs (100%) completed the four described stages. In 13% of the cases, the sequence was repeated more than once; of these, 8.7% returned to the genital positioning stage, while 4.35% restarted the process from the exploratory contact stage (Fig. 4).

### Male attraction to odours released by females

Males did not exhibit side preference in the blank test with *R. indica* teleiochrysalis females, and the time spent in each area did not differ, confirming the adequacy of the experimental setup (χ² = 0; DF = 1; *P* = 1; t = 0.10; DF = 18; *P* = 0.92).

**Fig. 5:**
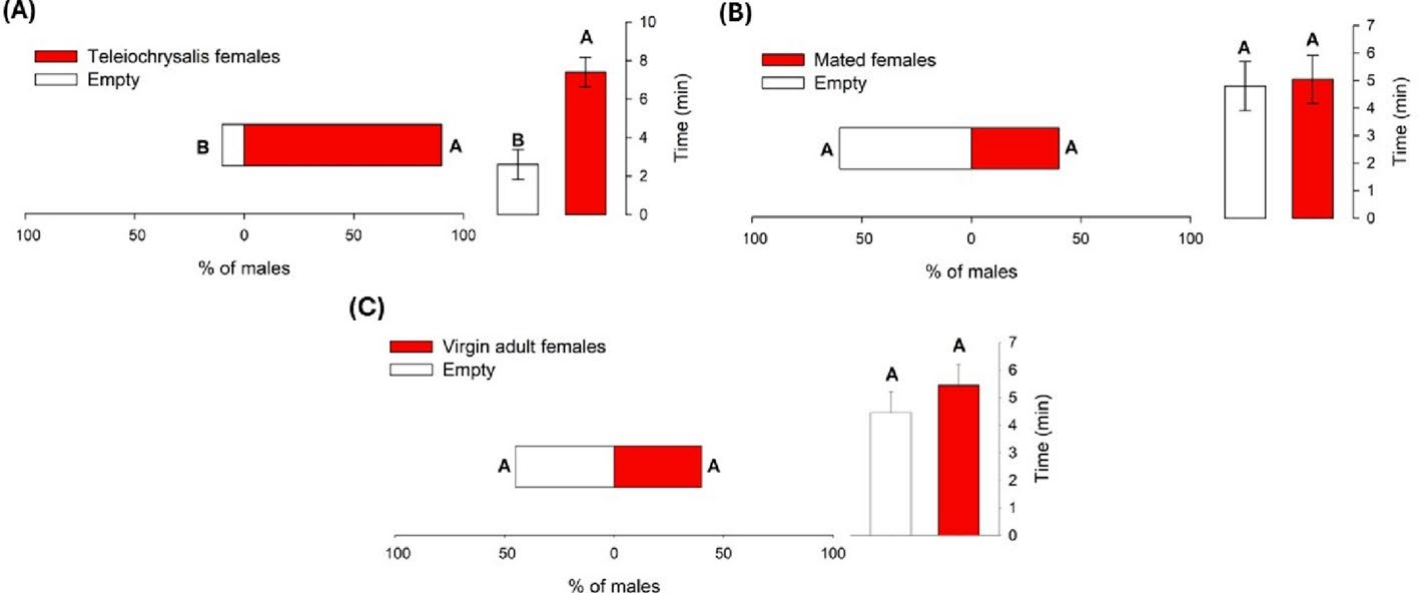
Percentage of *R. indica* males (*N* = 20) choosing between odours from teleiochrysalis females (**A**), mated females and (**B**) and virgin females (**C**) or an empty control, and mean residence time and standard error in each area. Percentages indicate the proportion of males that spent more time on each side of the arena. Different letters indicate significant differences according to the chi-square test (male percentage) and Student’s t-test (mean time)

In contrast, 80% of males chose to remain longer in the area containing the cage with teleiochrysalis females (χ² = 7.2; DF = 1; *P* = 0.007). The mean residence time in this area (7.47 min) was significantly higher than on the side with the empty cage (2.53 min) (t = 4.27; DF = 38; *P* = 0.0001) (Fig. 5A). However, when exposed to odours from recently mated females or virgin adult females, no significant differences were observed either in the proportion of responding males (mated: χ² = 0.8; DF = 1; *P* = 0.37; virgin: χ² = 0.06; DF = 1; *P* = 0.8) or in the mean residence time (mated: t = − 0.20; DF = 38; *P* = 0.84; virgin: t = 0.82; DF = 38; *P* = 0.42) (Fig. 5B and C).

### Male attraction to traces left by females

Males did not show a preference for either side of the experimental unit in the blank test with traces from virgin adult females of *R. indica*, and the time spent in each area did not differ, confirming the adequacy of the experimental setup (χ² = 0; DF = 1; *P* = 1; t = − 1.79; DF = 18; *P* = 0.09).

**Fig. 6:**
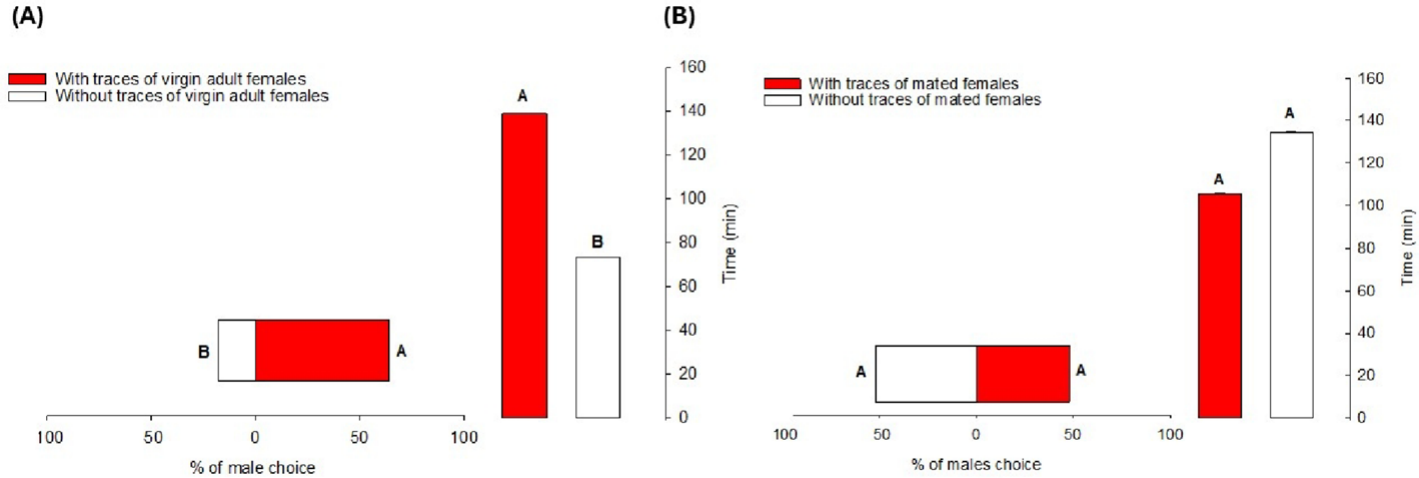
Percentage of choice of *R. indica* males (*N* = 20) when exposed to traces of virgin (**A**) or mated adult females (**B**) or to traces-free controls, and mean residence time and standard error in each area. Percentages indicate the proportion of males that spent more time on each side of the arena. Different letters indicate significant statistical differences according to the chi-square test (male percentage) and Student’s *t*-test (mean time)

The proportion of males that remained longer in the area containing traces from virgin adult females of *R. indica*, as well as the mean residence time, was significantly higher compared to the area without cues (χ² = 5.56; DF = 1; *P* = 0.02; t = 2.88; DF = 30; *P* = 0.007) (Fig. 6A). In contrast, when exposed to traces from mated females, no differences were detected either in male choice between sides or in mean residence time in the area with cues compared to the area without cues (χ² = 0.04; DF = 1; *P* = 0.83; t = − 0.85; DF = 38; *P* = 0.41) (Fig. 6B).

### Possibility of copulation between males and recently mated females

No copulation was observed between virgin males and recently mated females of *R. indica* during the 24-hour observation period, in 100% of the trials.

## Discussion

Although there are reports in the literature on mating-related behaviors of *R. indica*, this is the first study to provide a detailed description of this behavior, including the duration of each phase. The first phase observed was the approach of males and females with leg contact, followed by the characteristic pre-copulatory guarding position. The contact with the first pair of legs may indicate a tactile stimulus, which is an important cue for mate recognition in *T. urticae*, for example (Regev and Cone 1975). Haramoto (1966), while studying the mating behavior of mites of the family Tenuipalpidae (*Brevipalpus phoenicis* (Geijskes), *Brevipalpus californicus* (Banks), and *Tenuipalpus heveae* Baker), observed that after crawling beneath the female, the male partially positioned his body under hers. Furthermore, previous studies report that tenuipalpid males raise the opisthosoma until it touches the female’s genital opening, maintaining this position for 10 to 15 min while holding the female with their forelegs (Haramoto 1966; Manglitz and Cory 1953; Gerson 2008). In the present study, it was observed that *R. indica* males remained positioned behind the female for a significantly shorter period (48 s), subsequently bending the opisthosoma toward her body. Unlike what has been reported for other species in the family, *R. indica* females remained immobile, and the genital positioning phase (opisthosoma bending) showed a reduced duration (approximately 1 min). *R. indica* females expressed sexual receptivity by raising the opisthosoma and moving their hind legs, a behavior similar to that described in *T. urticae* mites (Schausberger et al. 2023). The copulation phase lasted on average 17.8 min, a value consistent with that reported by Hoy et al. (2007) for *R. indica*, cited as a personal observation. In species of the families Tetranychidae and Tydeidae, average copulation times are approximately 5 min and 2 min 43 s, respectively, when considering copulation with virgin females (Knop 1985; Rodrigues et al. 2017). This temporal difference among groups may be related to the amount of sperm transferred and/or to whether females require multiple copulations throughout their lifespan.

Males of *R. indica* preferred areas with teleiochrysalis females, whereas no preference was observed for areas with virgin or mated adult females. This pattern suggests that teleiochrysalis females emit stage-specific odors that facilitate mate location, although the precise nature of these odours (volatile or contact-borne) was not determined in the present study. As shown by Mizoguchi et al. (2003), female pheromones can be chemically characterized in mites, which reinforces the need for future analytical work to determine whether similar compounds occur in *R. indica*. Similar behavioral evidence for female-emitted sex pheromones has also been reported in other mite groups, supporting the hypothesis that female-produced signals mediate male attraction. (Więcek et al. 2025). In *T. urticae*, three processes have been identified as important for the detection of quiescent deutonymph females by males: tactile stimulation, the release of female sex pheromones, and cues associated with the webs produced by deutonymph females (Penman and Cone 1972). In the present study, only the selection of odours from teleiochrysalis females of *R. indica* by males was considered. Additionally, studies on *T. urticae* have shown that males are attracted to quiescent deutonymph females but not to protonymphs or active deutonymphs, and that their attraction response is more stable toward the end of the quiescent period (Cone et al. 1971a, b). Since *R. indica* males exhibit guarding behavior as a strategy to ensure the first copulation immediately after the female’s molt, it is expected that females become increasingly attractive as they approach ecdysis. On the other hand, males did not respond to odours from either recently mated females or virgin adult females. These results suggest that recently mated and virgin adult females of *R. indica* either do not release odours to attract males, release them in reduced quantities, or undergo changes in cue composition that prevent detection by males under the experimental conditions employed in this study. Differences in male sensitivity to female pheromones may alter mating outcomes and even lead to reproductive interference between species (Sato and Alba 2020). These results reinforce the view that pheromonal communication is behaviorally relevant across very different mite groups (Sonenshine 1985; Steidle et al. 2014).

An important methodological implication of these findings is that they argue against the possibility that male attraction was driven by plant-derived VOCs (Volatile_organic_compound) produced by feeding damage. The only treatment that elicited a significant behavioral response was the teleiochrysalis stage, during which females do not feed and therefore do not induce herbivory-related emissions. In contrast, no attraction was observed in treatments where females were actively feeding (virgin and mated adults). If feeding-induced plant volatiles were responsible for male orientation, the opposite pattern would be expected. This indicates that the male response is associated with cues specific to the teleiochrysalis stage, rather than with background plant volatiles generated during feeding.

Previous studies on arthropods have shown that when one or both sexes have already copulated, the attractiveness of females to males is reduced (AbdelöKader and Barak 1979; Elsayed 1990; Sanders and Lucuik 1972; Rasmy and Hussein 1994).

Males of *R. indica* responded to traces left by virgin adult females, spending more time in areas where these traces were present. However, they did not exhibit the same behavioral response to traces left by recently mated females. The chemical signals in traces differ from those emitted as odours in the arena assay. It is possible that odours are more reliable for detecting females than substrate traces (Schoonhoven et al. 2005). These results suggest that males of *R. indica* may have evolved mechanisms to recognize virgin females even in their physical absence, as a strategy to secure the first copulation. In mites of the genus *Tetranychus*, precopulatory guarding is considered an essential reproductive strategy, since only the first mating results in fertilization (Oku et al. 2015). However, there are still no reports on the number of copulations in mites of the genus *Raoiella*. Among mites, the detection of female reproductive status through chemical cues deposited on the substrate is well documented (Oku et al. 2005; Rodrigues et al. 2017). Studying *Tetranychus kanzawai* Oku et al. (2005) observed that males preferred traces left by virgin females over those left by mated females.

No mating attempts were observed between newly mated females of *R. indica* and virgin males during the 24-hour observation period. This outcome may be associated with the lack of male attraction toward recently mated females, possibly due to the absence of attractive odours emissions by these females. Alternatively, males may avoid mated females because their chances of reproducing are low: in several mite species, only the first copulation actually fertilizes the eggs, while subsequent matings do not result in offspring. In *T. urticae*, for instance, mated females have been reported to disperse after copulation, possibly as a strategy to avoid further male approaches and allocate energy toward oviposition (Oku 2010). Further studies are therefore needed to determine whether *R. indica* females engage in additional copulations after 24 h and whether they exhibit male-avoidance behaviors.

The results of this study provide the most detailed description to date of the mating behavior of *R. indica*, including the temporal characterization of its main phases, the behavioral signals involved, and the male response to chemical cues. The species exhibits a mating pattern broadly similar to that reported for other mites, but with unique features such as the short duration of genital positioning and copulation. Evidence suggests that male attraction is driven primarily by odours released by females in the quiescent stage (teleiochrysalis), which are absent or drastically reduced after copulation. This interpretation is consistent with studies in spider mites showing that mite-produced pheromones and host-plant kairomones can interact to shape orientation (Dicke 1986). Moreover, the ability of males to recognize traces left by virgin but not by mated females indicates a refined mechanism of sexual discrimination, likely adapted to the species’ single-fertilization system. The absence of mating with recently mated females further supports this hypothesis. Taken together, these findings make a significant contribution to understanding the behavioral ecology of *R. indica*.

The lack of male engagement in additional copulations with previously fertilized females may represent a reproductive strategy that minimizes the costs associated with multiple matings while maximizing the success of the already fertilized offspring. In tetranychid mites, for instance, although second copulations have been recorded, the costs of such interactions were demonstrated by Oku (2010), who reported reduced egg production in mated females when males were present, suggesting a fitness cost associated with male behavior.

Nevertheless, further experiments with intervals longer than 24 h after the first copulation are recommended to assess the potential for additional interactions and remating between virgin males and previously mated *R. indica* females. These findings highlight that chemical communication and the observed mating pattern are central to understanding the reproductive strategies of this species and contribute to broadening our knowledge of the behavioral ecology of mites in the family Tenuipalpidae.

## Supplementary Information

Below is the link to the electronic supplementary material.

Supplementary Material 1

## Data Availability

The experimental data of this study are available in https://doi.org/10.5281/zenodo.17414747.
